# Whose guns are stolen? The epidemiology of Gun theft victims

**DOI:** 10.1186/s40621-017-0109-8

**Published:** 2017-04-10

**Authors:** David Hemenway, Deborah Azrael, Matthew Miller

**Affiliations:** 1 Department of Health Policy and Management, Harvard TH Chan School of Public Health, 677 Huntington Avenue, Boston, MA 02115 USA; 2 Department of Health Sciences, Northeastern University, 360 Huntington Avenue, Boston, MA 02115 USA

**Keywords:** Firearms, Gun theft, Stolen guns

## Abstract

**Background:**

Gun theft is an important source of guns used by criminals. Yet no empirical work has focused on the characteristics of gun owners that distinguish those who have had their guns stolen from those who have not. In this study, we examine the demographics and behavioral characteristics of gun owners who report having had a gun stolen.

**Methods:**

Data come from a nationally representative probability-based online survey conducted in April 2015, with a linked follow-up survey in November 2015 that asked gun owners about any theft of their guns in the past 5 years.

**Results:**

Of 1,604 gun-owning respondents, 2.4% (95% CI 1.6,3.6) reported that one or more guns had been stolen, with a mean number of guns stolen per theft of 1.5 (95% CI 1.0,2.0]. Risk factors for having a gun stolen were owning 6 or more guns, owning guns for protection, carrying a gun in the past month, storing guns unsafely, and living in the South region of the United States. The South accounts for 37% of US households, 43% of gun owners, and two-thirds of all gun thefts.

**Conclusions:**

We estimate that there are approximately 250,000 gun theft incidents per year, with about 380,000 guns stolen. We find that certain types of gun owners-who own many guns, who carry guns, and who do not store guns safely-are at higher risk to have their guns stolen. Tracing data show that states in the South are exporters of crime guns used in other states. Our survey results find that the majority of guns stolen in the US come from the South.

## Background

Virtually every gun in the United States begins as a legal gun, manufactured legally and initially sold by a federally licensed gun dealer to an individual who passes a federal background check. However, many people with known anger, violence and/or alcohol problems can pass a federal background check (Swanson et al. 2015) and many people who cannot pass a background check still have easy access to firearms. The movement of guns to individuals who cannot pass a background check occurs via various mechanisms, including straw purchases, gifts, sales without a background check (Miller et al. 2017), and gun thefts.

Estimates over the past two decades suggest that 200,000 to 500,000 guns are stolen each year in the United States. Such estimates have come from several sources, including the National Crime Victimization Surveys (Langton 2012), police reports of stolen guns (DoJ 2012) and surveys of gun owners (Cook and Ludwig 1996).

The large number of guns stolen each year suggests that theft may be an important source of “crime guns.” Indeed, it appears that while gun theft is often not the proximate source of firearms for most criminals (Cook et al. 2015a; Chesnut et al. 2016), it is often the ultimate source-the way guns initially enter into the illegal market (Sheley and Wright 1993).

It is generally accepted that theft is an important source of guns for youth and criminals (Cook et al. 1995) and some research has examined how youth and offenders acquire their guns (Wright and Rossi 1986; Webster et al. 2002; Cook et al. 2015b). Yet as far as we can tell, there has not been a journal article in the peer-reviewed literature on the epidemiology of gun theft. And no study has identified characteristics of gun owners that distinguish those who have had guns stolen from those who have not.

What is currently known about the epidemiology of gun theft comes from Bureau of Justice Statistics reports summarizing results from the National Crime Victimization Surveys (NCVS) (Langton 2012; Zawitz 1995) NCVS data provide estimates of gun theft along with respondent demographic characteristics including household composition and US census region. An advantage of our survey over the NCVS is that we obtain much gun-related information about the respondent, such as how many guns are owned and whether the respondent carries a gun. Our survey allows us to compare gun owners whose guns were stolen with owners who did not experience a gun theft.

In this article, we use 2015 data from a large national survey of gun owners to describe gun theft victims-examining the characteristics of the gun owners whose guns have been stolen. In the jargon of epidemiology, we are describing some “risk factors” for being the type of person whose guns are stolen. We are not describing risk factors for being a gun thief.

## Methods

Data come from a nationally representative web-based survey conducted in April 2015, and a linked supplemental web-based survey, conducted in November 2015, of gun owners who responded to the initial survey. The authors designed the surveys, which were conducted by the survey firm Growth for Knowledge (GfK). The initial survey focused broadly on firearm ownership and use. Respondents (*n* = 3949) were drawn from GfK’s KnowledgePanel, an on-line panel of approximately 55,000 U.S. adults. The panel is selected on an ongoing basis to provide samples that are representative of the US population. Details of GfK’s survey design are described elsewhere (Miller et al. 2017; Betz et al. 2016; Knowledge Networks 2012; Azrael et al. 2015).

For the primary survey, 7,318 panel members received an invitation to participate. Of these, 3,949 completed the survey, yielding a survey completion rate of 55% (Callegaro and DiSogra 2008). Compared with survey non-respondents, respondents were more likely to be female, younger, less educated, unmarried and live in metropolitan areas.

Gun owners were identified through two questions: “Do you or does anyone else you live with currently own any type of guns?” followed by, among all respondents who answered in the affirmative: “Do you personally own a gun?”

In November 2015, GfK conducted a follow-up survey of the gun owners in the original survey (*n* = 2072 who were still in the GfK panel (*n* = 1880). Two questions were asked about gun theft: whether any of respondents’ guns were stolen in the past 5 years and how many were stolen.

Invitations to participate in the supplemental survey were sent by e-mail, and one reminder email was sent to non-respondents 3 days later. Participants did not receive any specific incentive to complete the survey, although GfK has a modest point-based incentive program through which participants accrue points for completing surveys and can later redeem them for cash, merchandise, or participation in sweepstakes.

Of those eligible for the supplemental survey (*n* = 1880), 1613 took the survey and 1604 answered the theft question (85%). Data for the outcome variable (i.e., having a gun stolen) come from this supplemental survey; all other data come from the individual responses to the original survey. The supplemental survey respondents did not differ from the gun-owning respondents in the primary survey with respect to age, gender, race, or type of gun most recently acquired.

To ensure reliable estimates about gun owners at the national level, the primary survey oversampled gun owners using GfK demographic profile variables. GfK provided survey weights for the current study that combined presample weights with study-specific poststratification weights to account for oversampling, and for survey nonresponse to both the April 2015 and November 2015 surveys.

The outcome variable is whether the gun owner had one or more guns stolen in the past 5 years. Explanatory variables include six demographic and five gun-related variables: (1) age (<30; 31–44; 45–59; 60+); (2) gender; (3) race/ethnicity (white non-hispanic or other); (4) household income (<$75,000 or $75,000+); (5) urbanicity (“Which best describes the community that you currently live in?”-rural, suburban, urban); (6) census region (Northeast, Midwest, South, West); (7) number of guns owned (<6 or 6+); (8) whether protection against people was one of the reasons for owning guns (9) carry guns (“In the past 30 days, have you carried a loaded handgun on your person” or not); (10) whether any guns were stored “in my car or other motor vehicle”; and (11) safe gun storage (worst = any gun loaded and unlocked; intermediate = at least one gun either loaded or unlocked; best = all guns unloaded and locked).

We conducted all analyses using Stata IC 14 (StataCorp), with use of appropriate weighting commands (using the weight variable provide by GfK) to generate national estimates and following the STROBE (Strengthening the Reporting of Observational Studies in Epidemiology) guidelines for reporting (von Elm et al. 2007). We use weighted percentages and calculate unadjusted odds ratios using logistic regression.

This work was supported by the Fund for a Safer Future and the Joyce Foundation. Funders did not play a role in the design, conduct, or reporting of the research, or in the decision to submit the manuscript for publication.

The Northeastern University Institutional Review Board approved the study.

## Results

Among gun owners, 2.4% report having one or more guns stolen in the past 5 years (Table 1). The mean number of guns stolen per incident was 1.5(95% CI: 1.0,2.0). Using data from the April 2015 survey, we estimated that 22% of US adults are gun owners (Azrael et al. 2015), consistent with findings from the General Social Surveys from the University of Chicago’s National Opinion Research Center (Smith and Son 2015). Using these results, and given approximately 242 million adults lived in the US (average for 2011–2015), we estimate that there were 1.2 million incidents of gun theft over a 5-year period, or about 250,000 incidents per year. With 1.5 guns stolen per incident, we estimate that approximately 380,000 guns (95% CI 260,000,510,000) were stolen per year.

**Table 1:** Correlates of having had one or more guns stolen (weighted)

	Number of Gun Owners (weighted)	% had guns stolen (95% CI)	Unadjusted Odds Ratios (95% CI)
Total	1604	2.4% [1.6,3.6]	
Demographic Characteristics			
Age			
< 30	184	2.1% [0.5,7.9]	Ref
30–44	366	4.3% [2.0,8.9]	2.1 [0.4,10.6]
45–59	507	2.1% [1.0,4.4]	1.0 [0.2,5.1]
60+	547	1.6% [0.9,2.9]	0.8 [0.2,3.6]
Gender			
Male	1159	2.2% [1.4,3.5]	Ref
Female	445	3.1% [1.3,6.8]	1.4 [0.5,3.7]
Ethnicity			
White (non- Hispanic)	1295	1.8% [1.1,2.8]	Ref
Non-White	309	5.2% [2.5,10.6}]	3.0 [1.2, 7.5]*
Income			
< $75,000	816	2.6% [1.5,4.7]	Ref
$75,000+	788	2.2% [1.3,3.9]	0.9 [0.4,2.0]
Urbanicity			
Rural	616	3.1% [1.6,5.8]	Ref
Suburban	732	1.6% [0.8,3.1]	0.5 [0.1,1.3]
Urban	248	3.4% [1.6,7.3]	0.9 [0.3,2.6]
Region			
Northeast	200	0.8% [0.2,3.2]	Ref
Midwest	383	1.2% [0.5,3.1]	1.5 [0.3,8.2]
South	692	3.7% [2.1,6.3]*	4.8 [1.1,21.6]*
West	329	2.1% [0.9,5.1]	2.7 [0.5,14.3]
Gun-Related Characteristics			
# Guns Owned			
< 6	1155	1.7% [1.0,2.9]	Ref
6+	410	4.5% [2.4,8.5]*	2.7 [1.1,6.4]*
Own Guns for Protection			
No	492	1.0 [0.3,2.2]	Ref
Yes	1121	3.1 [2.0,4.8]*	3.6 [1.3,10.0]**
Carry Guns			
No	1255	1.7% [1.0,2.8]	Ref
Yes	346	5.3% [2.8,9.7]**	3.3 [1.4,7.8]**
Store Gun in Car			
No	1519	2.2 [1.4,3.5]	Ref
Yes	94	5.7 [2.1,4.8]	2.6 [0.8,8.4]
Safe Gun Storage			
Worst	487	4.1 [2.4,7.0]	Ref
Intermediate	711	2.1 [1.0,4.4]	0.5 [0.2,1.3]
Best	374	1.0 [0.3,3.0]	0.2 [0.1,0.8]*

**significant at .01, * at .05

A significantly higher percentage of non-white gun owners had guns stolen (5.2 vs. 1.8%). Gun owners were also more likely to have guns stolen if they had six or more guns (4.5 vs. 1.7%), owned guns for protection (3.1 vs. 1.0%), carried guns in the past month (5.3 vs. 1.7%), did not store their guns in the safest manner (2.9 vs 1.0%) and, non-significantly, stored guns in the car (5.7 vs. 2.2%). A significantly higher percentage of gun owners from the South region had guns stolen (3.7 vs. 1.4%). Our survey indicates that 43% of gun owners reside in the South (694/1611) and 2/3 of gun thefts occur in the South.

## Discussion

Reporters are taught to investigate the who, what, how, when and why of an issue. This article focuses on one half of the who question-whose guns were stolen, but not who stole the guns. We believe ours is the first journal article whose primary focus is on any aspect of firearm theft.

Gun theft appears to be an important method by which guns enter the illegal market. Our estimate of 250,000 gun-theft incidents per year lies between older (1987–1992) and more recent estimates from the National Crime Victimization Surveys (Langton 2012; Zawitz 1995). Our finding that the mean number of guns stolen per incident is 1.5 is also consistent with NCVS findings (Langton 2012). We thus estimate that the total number of guns stolen annually is about 380,000, which is also generally consistent with previous findings (Langton 2012; Cook and Ludwig 1996; Zawitz 1995).

We find that white gun owners are substantially less likely to have guns stolen than non-white gun owners (the latter are a very heterogeneous group). National data show that whites are substantially less likely to be victims of both burglary and robbery than non-whites (US Department of Justice. Bureau of Justice Statistics. Crime Victims in the United States 2008). Although white gun owners have significantly lower rates of gun theft than non-white owners, since the large majority of gun owners are white (80%), most gun thefts (60%) involve white victims.

Our study is the first to explore the association between gun-related characteristics and gun theft victimization. We find that owning many guns, owning guns for protection, carrying guns, and storing guns unsafely are associated with having guns stolen. Storing guns in the car also appears to increase the risk; evidence suggests that many firearms are stolen from cars (Stolzenberg and D’Allessio 2000; Everytown for Gun Safety 2016; Freskos 2016). Owning many guns appears to be a risk factor for gun theft, perhaps because burglars see firearms as loot, so more household guns may make a more attractive target (Cook and Ludwig 2003). Carrying guns may increase the potential exposure to gun theft, and storing guns unlocked should make it easier for a thief to steal the gun.

We find that the majority of incidents of guns stolen in the United States come from one region-the South, a finding that is consistent with data from the NCVS (Langton 2012). Of the four main US regions, the South, accounts for 37% of households, 44% of the property crime (Federal Bureau of Investigation. Uniform Crime Reports 2014) but 2/3 of the gun theft incidents. The Southern region has the highest percentage of households with firearms and the least safe storage practices (Okoro et al. 2005). Not surprisingly, most Southern states are “exporters” of guns traced in crime (Mayors Against Illegal Guns 2010).

By contrast, our data show that the Northeast has lower rates of gun theft per gun owner, and lower levels of gun ownership, than the other three regions. States in the Northeast, particularly those with low levels of gun ownership and strong gun laws (e.g., Massachusetts, New York, Rhode Island, Connecticut) are “importers” of crime guns (Mayors Against Illegal Guns 2010).

This study has various limitations. First it relies exclusively on self-reports. Fortunately, our results concerning gun ownership, gun theft and other gun issues are largely consistent with those of other surveys (Azrael et al. 2015). Second, even though we obtain data from over 1600 gun owners, only 2.4% reported a gun theft in the previous 5 years, resulting in limited statistical power. Third we know almost nothing about the actual event–the type of gun stolen, where the gun was stored (e.g., at home, in the garage), whether it was locked up, the time of day or day of the week of the theft, who the thief was and whether he was known to the victim. The information we have about gun owners deals with responses at the time of the survey, not at the time of the theft or the exposure. For example, we do not know if respondents moved, or changed their firearms behaviors. Finally, we have no estimate of the number of guns stolen from juveniles, from individuals who were not gun owners at the time of the survey, or from gun manufacturers, wholesalers or licensed retailers (over 6 thousand guns were reported stolen from licensed dealers in 2015, and another 8 thousand lost) (Bureau of Alcohol, Tobacco, Firearms, and Explosives. (ATF) 2016). Indeed, we do not know whether or not any of the respondents in our survey are gun dealers (though the question on gun theft referred to their personal firearms).

Hundreds of thousands of guns are stolen each year; gun theft is an important way that guns enter the illegal market. There are many ways gun theft could be reduced. Personalized guns would limit the utility of firearms to unauthorized users and reduce the incentive to steal guns. In addition, if gun owners stored their guns more safely, probably fewer guns would be stolen. Changes in how owners store their guns could occur from changes in laws and in social norms concerning gun storage (Everytown for Gun Safety 2016). Physicians, gun shops, gun trainers and others could help change norms concerning storage practices. Gun theft could also be curtailed by improvements in gun storage technology that reduce the price while making it quicker for the authorized user, and harder for the thief, to gain access to the firearm. Law enforcement could help by increasing the likelihood of investigating and prosecuting gun theft and by disrupting the stolen gun market.

Unfortunately, there has been limited funding for firearms research resulting in insufficient firearm research compared to the size of the public health problem (Stark and Shah 2017), and relatively little is known about gun theft. For example, we could not find any journal articles on the relationship between gun storage and gun theft in the United States. Our survey had only a few questions about gun theft; it would be informative to have a large survey with a major focus on gun theft in order to provide information about the what, where, why and how of the event. Gun theft is an important issue that deserves more academic attention.

## Conclusions

A common way guns get into criminal hands appears to be through gun theft. Yet little is known about gun theft in the United States; ours appears to be the first journal article focusing on gun theft. We estimate that approximately 380,000 guns are stolen each year, in about 250,000 incidents. Gun owners who own more guns, own them for protection, carry guns, store gun unsafely, and in cars, are more likely to have guns stolen. Although the Southern region is home to 37% of US households, approximately two-thirds of guns stolen are from the South. Attempts to reduce the number of stolen guns may do well to focus on individuals and places where most gun theft occurs.
